# Efficacy of continuous epidural infusion with epidural electric stimulation compared to that of conventional continuous epidural infusion for acute herpes zoster management: a retrospective study

**DOI:** 10.1186/s12871-020-0950-0

**Published:** 2020-01-28

**Authors:** Chung Hun Lee, Sang Sik Choi, Mi Kyoung Lee, Yeon Joo Lee, Jong Sun Park

**Affiliations:** Department of Anesthesiology and Pain Medicine, Korea University Medical Center, Guro Hospital, Gurodong Road 148, Guro-Gu, Seoul 08308 Republic of Korea

**Keywords:** Herpes zoster, Postherpetic neuralgia, Epidural analgesia, Continuous epidural infusion, Electric stimulation

## Abstract

**Background:**

Continuous epidural infusions are commonly used in clinical settings to reduce the likelihood of transition to postherpetic neuralgia via pain control. The purpose of this study was to compare the efficacy of conventional continuous epidural infusion to that of continuous epidural infusion in which the catheter is guided by electric stimulation to areas with neurological damage for the treatment of zoster-related pain and prevention of postherpetic neuralgia.

**Methods:**

We analyzed the medical records of 114 patients in this study. The patients were divided into two groups: contrast (conventional continuous epidural infusion) and stimulation (continuous epidural infusion with epidural electric stimulation). In the contrast group, the position of the epidural catheter was confirmed using contrast medium alone, whereas in the stimulation group, the site of herpes zoster infection was identified through electric stimulation using a guidewire in the catheter. Clinical efficacy was assessed using a numerical rating scale (pain score) up to 6 months after the procedures. We compared the percentage of patients who showed complete remission (pain score less than 2 and no further medication) in each group. We also investigated whether the patients required additional interventional treatment due to insufficient pain control during the 6-month follow-up period after each procedure.

**Results:**

After adjusting for confounding variables, the pain score was significantly lower in the stimulation group than in the contrast group for 6 months after the procedure. After adjustment, the odds of complete remission were 1.9-times higher in the stimulation group than in the contrast group (95% confidence interval [CI]: 0.81–4.44, *P* = 0.14). Patients in the contrast group were significantly more likely to require other interventions within 6 months of the procedure than patients in the stimulation group (odds ratio: 3.62, 95% CI: 1.17–11.19, *P* = 0.03).

**Conclusion:**

Epidural drug administration to specific spinal segments using electric stimulation catheters may be more helpful than conventional continuous epidural infusion for improving pain and preventing postherpetic neuralgia in the acute phase of herpes zoster.

## Background

Herpes zoster is caused by reactivation of the latent varicella zoster virus (VZV), which persists in the dorsal root ganglion after the initial infection. It causes irritation along the nerve distribution, abnormal sensitization of nociceptive receptors, and induces hyperactivity of the central nerve [1]. The frequency of reactivation increases particularly in elderly patients and in patients with inhibited virus-specific cellular immunity [2, 3]. The acute phase of herpes zoster is defined as the period within 30 days of rash onset [4]. Postherpetic neuralgia (PHN) is the most common complication of herpes zoster and can occur if the patient is not properly treated during the acute phase; in elderly patients, it can be due to a weakened immune system, despite proper treatment [5]. PHN is a neuropathic condition associated with severe refractory pain, and it lowers the quality of life. Among patients with herpes zoster, 70% of those over 50 years of age complained of pain 1 month following the disappearance of the skin rash, whereas 50% of patients aged 70 years or older experienced pain 1 year following the disappearance of the rash [6, 7]. Patients at risk of developing PHN may therefore require aggressive treatment using appropriate drug therapies.

Epidural, sympathetic, and paravertebral blocks are considered active treatments for acute episodes of herpes zoster. For acute herpes zoster, continuous epidural infusion is commonly used in clinical settings and is reported to reduce the likelihood of transition to PHN via pain control [8–10]. For conventional continuous epidural infusion, the location of the epidural catheter is confirmed by injecting a contrast agent at the suspected epidural level, which is identified based on the site of rash or pain.

To improve the efficacy of continuous epidural infusion, it is important that the drug is administered precisely at the epidural level of the herpes zoster infection [11, 12], which is dependent on the proximity of the catheter to the affected nerve site. For this purpose, a continuous epidural infusion is administered utilizing epidural electric stimulation to confirm whether the catheter has accurately reached the site of infection. The present retrospective study was designed to assess the efficacy of continuous epidural infusion utilizing epidural electric stimulation, in comparison to that of conventional epidural infusion, for the control of acute herpes zoster pain and prevention of PHN.

## Methods

### Study design

Our retrospective observational study adhered to the STROBE checklist (S1 checklist) and was approved by the institutional review board of our hospital (No: 2019GR0073, March 11, 2019).

The medical records of patients who underwent continuous epidural infusion for herpes zoster-related pain from June 2010 to October 2017 were collected. Among these, only the medical records of patients who received continuous epidural infusion within 30 days of rash onset were included. The patients were divided into two groups depending on the type of epidural catheter used for continuous epidural infusion: the contrast group, which used standard epidural catheters, and the stimulation group, which used epidural catheters with electric stimulation. Patients had to meet the following criteria to be included in the present study: those older than 50 years of age with a numeric rating scale (NRS) score of 4 or greater; those who had only received standard drug therapy, including antiviral agents, until the administration of continuous epidural infusion; and those who underwent follow-up for a period of 6 months after continuous epidural infusion. The following criteria were used for exclusion: patients with insufficient medical records; patients who received other drugs (such as opioids) with epidural catheters; patients with immunosuppressed status; patients in whom the catheter was not maintained for more than 10 days after continuous epidural infusion; patients who did not receive standard medication (such as antiviral agents) until the procedure was performed; and patients who discontinued the prescribed standard medication (anticonvulsants and analgesics) because of adverse effects. Additionally, patients who had undergone other interventional procedures due to the exacerbation of herpes zoster-related pain within 6 months of continuous epidural infusion were excluded, and the requirement rates for interventional procedures were analyzed separately.

### Procedure

#### Continuous epidural infusion: the contrast group

**Fig. 1:**
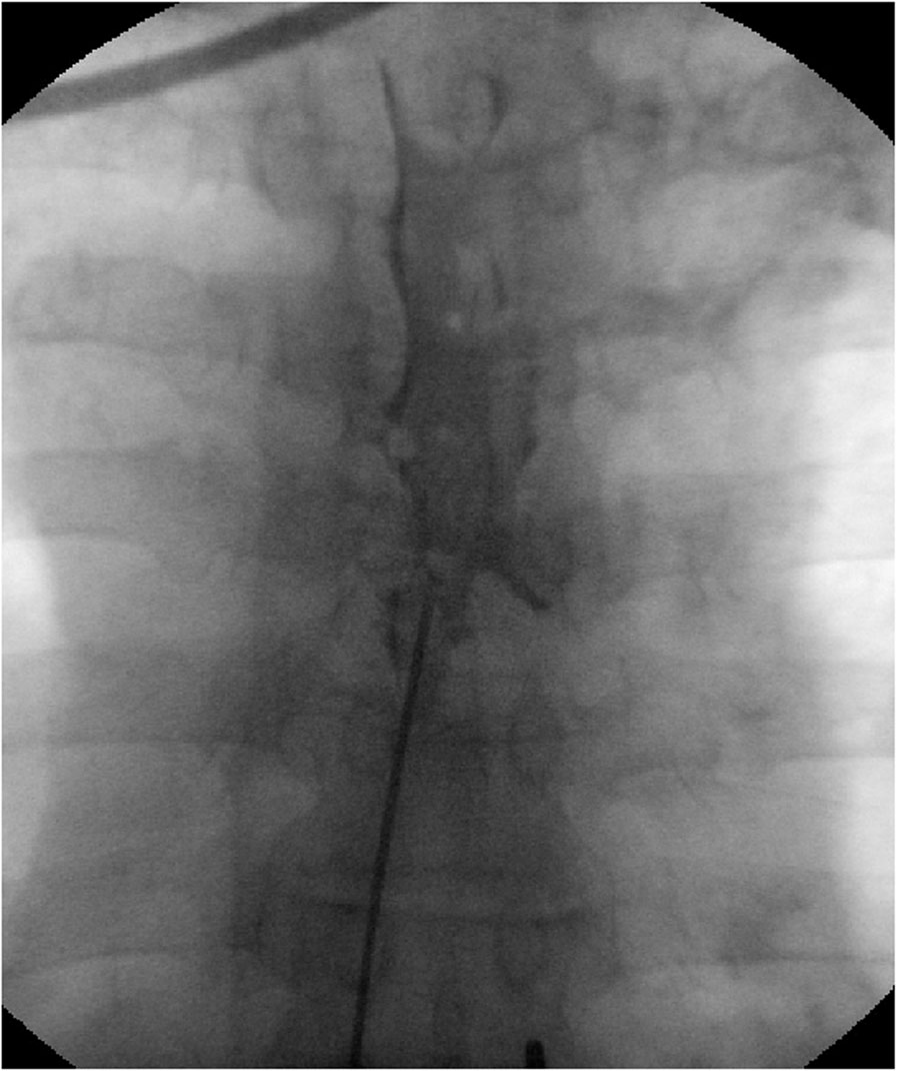
Fluoroscopic images of conventional continuous epidural block. The position of the catheter tip was confirmed using a contrast agent

Patients were placed in the prone position, and an aseptic dressing was applied to the procedure site. An 18-gauge Tuohy needle was inserted into the inter-laminar space three levels below the target level under the guidance of fluoroscopic imaging. The loss of resistance (LOR) technique was used to verify whether the Tuohy needle was placed in the epidural space. After confirming the epidural space, a 20-gauge epidural catheter (Perifix® Soft Tip epidural anesthesia catheter, B. Braun, Melsungen, Germany) was inserted through the Tuohy needle, and diffusion of the contrast agent was checked to ensure that the catheter was placed in the proper position (Fig. 1). Once the epidural catheters were confirmed to have been placed at the epidural levels identified based on the site of pain and rash, 0.187% ropivacaine and 1 mg dexamethasone (8 mL total) were administered via the epidural catheter.

#### Continuous epidural infusion: the stimulation group

**Fig. 2:**
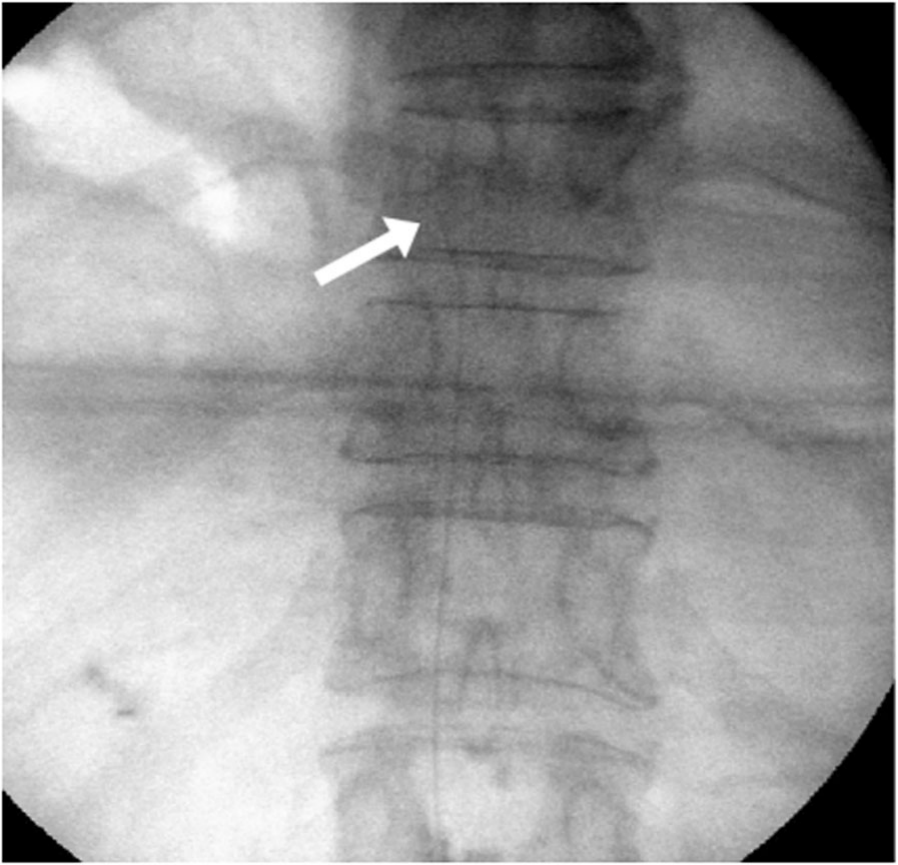
Fluoroscopic images of continuous epidural block using the EpiStim catheter. This catheter has a built-in conductive guidewire that allows the detection of the location of the catheter tip using radiography along with electric stimulation. The arrow indicates the guidewire in the EpiStim catheter

Similar to that in the contrast group, after the epidural space was identified using the LOR technique, a 20-gauge epidural catheter (EpiStim™, length = 800 mm, Sewoon Medical Co., Ltd., Seoul, Korea) allowing radiographic confirmation was placed at the target level through the Tuohy needle (Fig. 2). This type of epidural catheter has a built-in conductive guidewire (Nitinol, length = 1100 mm) with 800 mm inside the catheter and 300 mm exposed for connection to an electric nerve-stimulator. The cathode of the electric nerve stimulator (Life-Tech EZstim, Stafford, TX, USA) was connected to the exposed guidewire, and the anode was attached to an electrode on the patient’s calf. A 0–5 mA electric current was then delivered through the guidewire. Verbal communication with the patient confirmed that the electric stimulation had reached the herpes zoster-affected area. The catheter was placed in the appropriate epidural space, and once electric stimulation had been initiated, verbal communication with the patient confirmed the sensation. After patient confirmation, further communication established that the electric stimulus was following the herpes zoster dermatome. However, if electric stimulation was observed in a region other than the herpes zoster dermatome, the epidural catheter was adjusted under fluoroscopy, and electric stimulation was repeated to confirm localization to the herpes zoster-affected area. Once the stimulus effectively reached the herpes zoster-affected area, the guidewire was removed from the epidural catheter. The position of the epidural catheter tip was confirmed with a contrast agent under a fluoroscope. Subsequently, 0.187% ropivacaine and 1 mg of dexamethasone (8 mL total) were administered via the catheter.

In both groups, the epidural catheter was fixed by subcutaneous tunneling to decrease the risk of infection and catheter migration. The inserted catheter was maintained in its position for a minimum of 10 days and was removed after 2 weeks. While patients were undergoing catheterization as inpatients and outpatients, a physician changed dressings daily and monitored the procedure site.

After the initial drug injection, patients in both groups were administered a continuous epidural infusion (275 mL) of 0.11–15% ropivacaine at a rate of 4 mL/h using a portable balloon infusion device (AutoFuser pump, ACE Medical Co., Ltd., Seoul, Korea), for the entire duration of catheter insertion. Ropivacaine concentrations were adjusted according to the degree of pain relief or side effects. Additionally, anticonvulsants (pregabalin or gabapentin) and analgesics were administered to patients in both the groups. Anticonvulsants were prescribed by adjusting the drug dose according to age and renal function and were tapered according to symptoms. Oxycodone was administered as an analgesic starting with the minimum reported effective dose for PHN [13].

### Data collection

Data on age, sex, involved dermatome, and days from the onset of rash to continuous epidural infusion, as well as history of hypertension, diabetes, liver disease, kidney disease, asthma, and amount of ropivacaine in the infusion device were collected. Pain was assessed with a pain score using an 11-point verbal NRS (0 = no pain, 10 = unbearable pain). Pain score data were collected from the patients’ medical records at different time points: at baseline (immediately before the procedure) and immediately after, 14 days after, and 1, 3, and 6 months after the procedure. Complete remission was defined as a pain score of less than 2 with no further medication required. The number of patients included in this category during the 6-month follow-up period was also recorded. Finally, we also investigated whether other interventional treatments were required due to insufficient pain control during the 6-month follow-up period after each procedure.

### Outcome measures

We compared the baseline pain scores of both groups and assessed whether they were significantly reduced during the 6-month post-procedure period. To assess analgesic effects, we compared the pain scores of the two groups at baseline and immediately after, 14 days after, and 1, 3, and 6 months after the procedure, following correction for confounding variables. Additionally, we compared the percentage of patients who achieved complete remission in each group and the proportion of patients requiring additional nerve block due to inadequate pain control after each procedure.

### Statistical analysis

Demographic data were assessed for normality using the the Kolmogorov-Smirnov test. Comparisons between the groups were made using an independent t-test for normally distributed variables, and non-normally distributed variables were compared using the Mann-Whitney U test. A repeated measures analysis of variance with the Bonferroni post-hoc test was used to determine whether the pain score was significantly reduced at each time point after the procedure compared to that at the baseline within each treatment group. After correcting for various confounding variables (e.g. age, sex, site of herpes zoster infection, history of hypertension, diabetes mellitus, asthma, hepatic disease, and kidney disease), we analyzed the differences in pain scores between the groups using covariance analysis. Between the two groups, multivariable logistic regression analysis was used to compare the odds of achieving complete remission and the odds of undergoing other interventional procedures within 6 months of the procedure. Data were reported as mean ± standard deviation or median (interquartile range) and were analyzed with the Statistical Package for the Social Sciences software (version 17.0, IBM, Chicago, IL, USA). All statistical tests were two-tailed, and the threshold for statistical significance was set at *P* < 0.05.

## Results

**Fig. 3:**
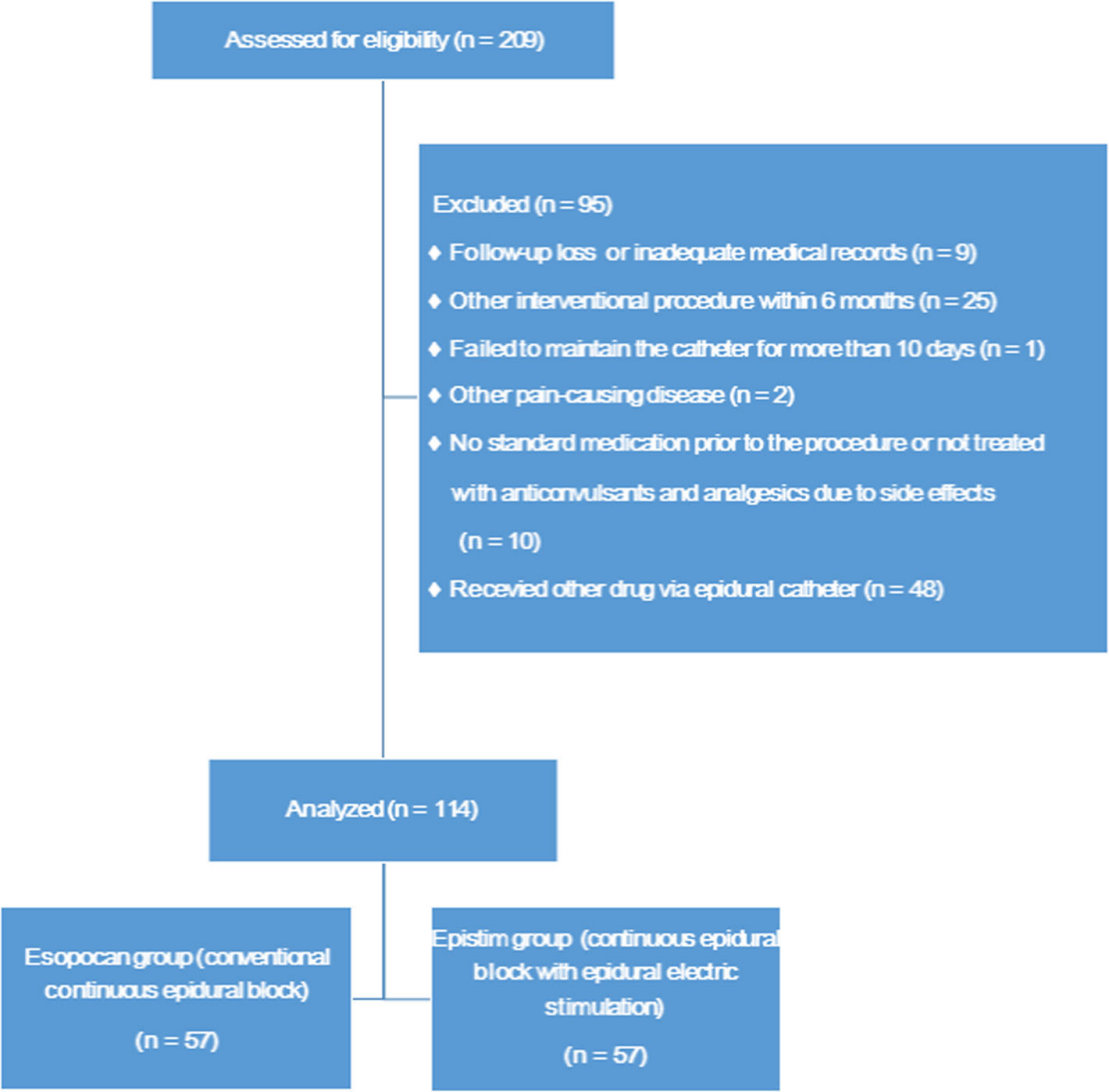
Flow diagram showing patient inclusion

We reviewed 209 patient records. Nine patients missed follow-up appointments or had inadequate medical records for the 6 months following the procedure. Twenty-five patients underwent other interventional procedures within 6 months of continuous epidural infusion. In one patient, the catheter could not be maintained for more than 10 days due to side effects associated with continuous epidural infusion. Two patients did not receive antiviral drugs at the beginning of the herpes zoster episode. During the 6-month follow-up period, two patients reported other painful diseases. The medical records of these patients were excluded from the final analysis. Additionally, eight patients stopped using anticonvulsants and analgesics due to drug-associated side effects after the procedure, and 48 patients received other drugs, such as opioids, via the epidural catheter. To prevent drug-induced bias, these patients were also excluded from the final analysis. Finally, the medical records of 114 patients were analyzed, with 57 patients each in the contrast and stimulation groups (Fig. 3).

There were no significant differences in baseline demographics between the groups (Table 1). Bonferroni post-hoc tests revealed that the pain scores at each time point after the procedure were significantly lower than those at baseline in both groups (Table 2).

**Table 1:** Baseline characteristics of the patients

	Acute HZ (≤ 30 days)		*P* value
	Contrast group (*n* = 57)	Stimulation group (*n* = 57)	
Age (years)	67.0 ± 10.3	66.2 ± 11.7	*P* = 0.72
Sex (M/F)	25/32	16/41	*P* = 0.12
Site of HZ infection	C: 14	C: 13	*P* = 0.52
	T: 36	T: 33	
	L: 7	L: 11	
HTN	26 (46% [33, 58%])	21 (37% [26, 50%])	*P* = 0.45
DM	11 (19% [11, 31%])	16 (28% [18, 41%])	*P* = 0.38
Asthma	3 (5% [2, 14%])	1 (2% [0, 9%])	*P* = 0.62
Hepatic disease	5 (9% [4, 19%])	3 (5% [2, 14%])	*P* = 0.72
Kidney disease	2 (4% [1, 12%])	3 (5% [2, 14%])	*P* = 1.0
Avg. amount of ropivacaine in the infusion device (ml)	37.7 ± 3.8	37.5 ± 4.0	*P* = 0.79
Baseline pain score^a^	8 (7–8)	8 (7–8)	*P* = 0.22

Data are represented as mean ± standard deviation, median (interquartile range), or number (% [95% confidence interval])

*HZ* herpes zoster, *HTN* hypertension, *DM* diabetes mellitus, *Avg* average, ^a^Pain score on an 11-point (0–10) numerical rating scale, *C* cervical, *T* thoracic, *L* lumbar

**Table 2:** Comparison with baseline pain scores at each time point

Group		Contrast group		Stimulation group	
Period A	Period B	Average difference (a − b)	*P* value	Average difference (a − b)	*P* value
Baseline pain score^a^	(1)	3.97	*P* < 0.001	4.25	*P* < 0.001
	(2)	4.62	*P* < 0.001	5.51	*P* < 0.001
	(3)	4.47	*P* < 0.001	5.21	*P* < 0.001
	(4)	4.88	*P* < 0.001	5.65	*P* < 0.001
	(5)	5.25	*P* < 0.001	5.88	*P* < 0.001

^a^Pain score on an 11-point (0–10) numerical rating scale. (1) Pain score immediately after epidural procedure, (2) Pain score 14 days after epidural procedure, (3) Pain score 1 month after epidural procedure, (4) Pain score 3 months after epidural procedure, (5) Pain score 6 months after epidural procedure. Data were analyzed using the Bonferroni post-hoc test. *P* value < 0.01 was considered statistically significant

When the post-procedure pain scores of the two groups were compared, (after correcting for confounding variables), no significant differences were observed immediately after the procedure; however, there were significant differences in the post-procedure pain scores between the two groups after the 14th day and up to the 6th month (Table 3).

**Table 3:** Comparison of pain scores between the groups after correction for confounding variables

	Acute HZ (≤ 30 days)		*P* value
	Contrast group (*n* = 57)	Stimulation group (*n* = 57)	
Baseline pain score	7.4 ± 1.5	7.2 ± 1.5	*P* = 0.28
Pain score^a^ immediately after epidural procedure	3.5 ± 2.0	2.9 ± 1.8	*P* = 0.25
Pain score^a^ 14 days after epidural procedure	2.8 ± 1.9	1.7 ± 0.8	*P* = 0.001
Pain score^a^ 1 month after epidural procedure	3.0 ± 2.1	1.9 ± 1.1	*P* = 0.01
Pain score^a^ 3 months after epidural procedure	2.6 ± 1.8	1.5 ± 1.2	*P* = 0.001
Pain score^a^ 6 months after epidural procedure	2.2 ± 1.8	1.3 ± 1.1	*P* = 0.01

Data are reported as adjusted mean ± standard deviation. Data were analyzed for the difference in pain scores between the groups using covariance analysis. Adjustments were made for age, sex, timing from rash to epidural procedure, location of herpes zoster, hypertension, diabetes mellitus, asthma, hepatic disease, and kidney disease

*HZ* herpes zoster, ^a^Pain score on an 11-point (0–10) numerical rating scale

The difference between the post-procedure pain scores of the two groups was analyzed at each level. The cervical and thoracic level results were similar to those of the entire analysis, showing better pain control in the stimulation group than that in the contrast group. However, there was no significant difference between the two groups in the lumbar area, which was an affected site for a small number of patients (Table 4).

**Table 4:** Comparison of pain scores between the groups for cervical, thoracic, and lumbar levels

	Cervical area			Thoracic area			Lumbar area		
	Acute HZ (≤ 30 days)		*P* value	Acute HZ (≤ 30 days)		*P* value	Acute HZ (≤ 30 days)		*P* value
	Contrast group (*n* = 14)	Stimulation group (*n* = 13)		Contrast group (*n* = 36)	Stimulation group (*n* = 33)		Contrast group (*n* = 7)	Stimulation group (*n* = 11)	
Baseline pain score	7.8 ± 1.4	6.9 ± 1.3	*P* = 0.13	7.4 ± 1.6	7.2 ± 1.5	*P* = 0.85	7.1 ± 1.7	7.3 ± 2.0	*P* = 0.9
Pain score^a^ immediately after epidural procedure	3.8 ± 2.0	3.0 ± 2.1	*P* = 0.44	3.5 ± 2.0	3.1 ± 1.7	*P* = 0.51	3.0 ± 1.8	2.1 ± 1.8	*P* = 0.58
Pain score^a^ 14 days after epidural procedure	3.2 ± 2.1	1.7 ± 0.8	*P* = 0.02	2.8 ± 1.9	1.7 ± 0.9	*P* = 0.03	2.3 ± 1.0	1.5 ± 0.8	*P* = 0.08
Pain score^a^ 1 month after epidural procedure	3.4 ± 2.5	1.9 ± 1.2	*P* = 0.06	2.9 ± 2.1	2.0 ± 1.1	*P* = 0.19	2.7 ± 1.5	1.6 ± 1.1	*P* = 0.12
Pain score^a^ 3 months after epidural procedure	2.3 ± 1.3	1.3 ± 1.3	*P* = 0.04	2.6 ± 2.0	1.6 ± 1.1	*P* = 0.04	2.9 ± 2.0	1.3 ± 1.2	*P* = 0.07
Pain score^a^ 6 months after epidural procedure	2.2 ± 1.8	1.2 ± 1.1	*P* = 0.03	2.2 ± 1.8	1.3 ± 1.1	*P* = 0.08	2.3 ± 1.6	1.2 ± 1.2	*P* = 0.46

Data are represented as adjusted mean ± standard deviation. Data were analyzed for differences in pain score between the groups using covariance analysis. Adjustments were made for age, sex, timing from rash to epidural procedure, hypertension, diabetes mellitus, asthma, hepatic disease, and kidney disease

*HZ* herpes zoster, ^a^Pain score on an 11-point (0–10) numerical rating scale

The odds of complete remission were 1.90-times higher in the stimulation group than in the contrast group (Table 5).

**Table 5:** Comparison of complete remission between the contrast and stimulation groups during the 6-month follow-up period after each procedure

	Contrast Group	Stimulation Group	Adjusted OR (95% CI) Reference: contrast group	*P* value
Acute HZ (≤3 0 days)	29/57 [51% (38, 63%)]	41/57 [72% (59, 82%)]	1.90 (0.81–4.44)	*P* = 0.14

Complete remission is defined as a pain score of less than 2 with no further medication. Data are represented as number (% [95% confidence interval]) and were analyzed by logistic regression analysis. Adjustments were made for age, sex, location of herpes zoster, days from the onset of rash to procedure, hypertension history, diabetes mellitus history, asthma history, hepatic disease history, kidney disease history, and baseline pain score

*HZ* herpes zoster, *OR* odds ratio, *CI* confidence interval

The odds of undergoing other interventional procedures within 6 months of continuous epidural infusion due to insufficient pain control were 3.62-times higher in the contrast group than in the stimulation group (Table 6).

**Table 6:** Comparison of implemented procedures due to insufficient pain control during the 6-month follow-up period

	Contrast Group	Stimulation Group	Adjusted OR (95% CI) Reference: stimulation group	*P* value
Acute HZ (≤30 days)	20/77 [26% (17, 37%)]	5/62 [8% (3, 18%)]	3.62 (1.17–11.19)	*P* = 0.03

Data are reported as number [% (95% confidence interval)]. Data were analyzed by logistic regression analysis. Adjustments were made for age, sex, location of herpes zoster, days from the onset of rash to procedure, hypertension history, diabetes mellitus history, asthma history, hepatic disease history, kidney disease history, and baseline pain score

*HZ* herpes zoster, *OR* odds ratio, *CI* confidence interval

## Discussion

The purpose of the present study was to evaluate whether a procedure confirming a nerve block at the site of herpes zoster infection by application of epidural electric stimulation was more effective in reducing pain and preventing PHN than a procedure that identifies the location of epidural catheters with contrast agents alone.

In the present study, the pain scores of patients in both tested groups were significantly lower over the 6-month follow-up period than at the baseline. From 14 days to 6 months after the procedure (follow-up period), pain scores were significantly lower in the stimulation group than in the contrast group. The odds of complete remission of herpes zoster up to 6 months after the procedure was 1.9-times higher in the stimulation group than in the contrast group. This suggests that administering the drug after confirming the correct VZV-containing dorsal root ganglion using epidural electric stimulation may be more effective in treating herpes zoster than the conventional continuous epidural infusion. The proportion of patients who received other epidural blocks because of the lack of pain control within the 6 months following the procedure, was approximately one-third lower in the stimulation group than that in the contrast group.

There was also a difference in the drug injection site of the epidural catheter tip between the two groups. Reportedly, closed-tip, multi-orifice catheters are more effective for sensory blocks than open-tip, end-hole catheters. However, in our study, the stimulation group (in which open-tip, end-hole catheters were used) showed greater pain reduction than the contrast group (in which closed-tip multi-orifice catheters were used) [14, 15]. These results suggest that a continuous epidural infusion utilizing electric stimulation to confirm the location of herpes zoster is more effective in achieving pain relief than the conventional continuous epidural infusion. EpiStim™ epidural catheters have a bent tip and a flexible guidewire and use electric stimulation to identify the affected area, increasing the maneuverability of the catheter and making it easier to position the catheter at the target site [12]. These features yielded significant differences between the contrast and stimulation groups in our results.

In our study, there was no significant difference in pain reduction immediately after the procedure between the two groups. This is likely due to the spread of 8 mL of drug epidurally administered during the procedure. After administration, it is likely that the drug spread to adjacent dermatomes. Therefore, even if the epidural catheter was not precisely at the affected site, the drug may still have spread to the site of the herpes zoster infection, but this would occur only with a single epidural block. When the drug was administered continuously at the rate of 4 mL/h via a portable infusion pump, the spread of the drug decreased considerably. Therefore, precise administration of the drug to the correct site would have been possible only if the catheter was positioned in close proximity to the herpes zoster infection site. We suggest that the differences in pain scores at 14 days and 1, 3, and 6 months after the procedure were attributable to continued pain relief, despite reduced drug efficacy over the period of continuous administration, if the catheter was correctly placed in the target region.

Due to the complexity of the pathophysiological mechanisms that contribute to the progression of acute herpes zoster to PHN, various preventive strategies have been proposed, including vaccinations and the use of antiviral agents, anticonvulsants, and corticosteroids. However, according to a recent systematic review and meta-analysis, the efficacy of these treatments in preventing PHN is limited [16–21]. We focused on the nerve damage caused by VZV for the treatment of acute herpes zoster and PHN prevention. Reactivated VZV in the dorsal root ganglion, which manifests as herpes zoster, subsequently diffuses to the affected dermatome producing an inflammatory response and inducing nerve damage. Severe initial nerve damage or the inability to regain normal function after the loss of nerve function can lead to PHN [22]. Therefore, proactive treatment before nerve injury can help prevent PHN. According to a recent meta-analysis, continuous epidural infusion in acute herpes zoster is effective in preventing PHN [9]. The rationale behind the application of epidural blocks to control acute herpes zoster pain and prevent PHN is that the discontinued delivery of an invasive afferent stimulus to the central nervous system and improved flow of blood to the subjects’ nerve tissue will minimize neural damage and reduce sensitization. In addition, it is possible that local anesthetics, along with the anti-inflammatory effects of corticosteroids, could be effective in areas corresponding to the affected nerves [23]. Epidural administration of steroids not only inhibits inflammation but also reduces deafferentation by decreasing any neural ischemia resulting from inflammatory swelling [21]. Local anesthetics administered epidurally control pain and interfere with sensitization by blocking sympathetic nerves; however, to maximize the effects of epidural steroids and local anesthetics on the affected site, it is important to administer the drug precisely to the site of nerve injury [24]. Therefore, we performed epidural electric stimulation to specifically identify the site sustaining the nerve injury caused by herpes zoster. This method allows for more accurate catheter placement than the conventional method, where the diffusion image of a contrast agent is used to confirm the location of the catheter.

In the current study, patients who could not maintain the inserted continuous epidural catheter for more than 10 days were excluded from the analysis because according to a previous study, a single epidural block may be effective in controlling herpes zoster-related pain, but it has limited efficacy in the prevention of PHN [25, 26].

All the patients included in our study underwent continuous epidural infusion and simultaneously took anticonvulsants and analgesics. To avoid bias due to drug treatments, patients who discontinued the drug due to side effects from other treatments and those who were administered drugs other than local anesthetics and steroids via the epidural catheter, such as opioids, were excluded from the analysis.

The complete remission rate in the present study was 51% in the contrast group and 72% in the stimulation group. Reportedly, the greater the severity of acute herpes zoster pain, the greater the likelihood of its progression to PHN [5, 27]. In our clinic, invasive treatments, such as continuous epidural infusion, are not performed for less severe cases of herpes zoster (pain score, < 4). Consequently, all the participants in the present study had pain scores of 4 or higher (mean 7.5 ± 1.5 and 7.1 ± 1.4 in the control and stimulation groups, respectively), which may be one of the reasons for the lower rates of complete remission. Additionally, the definition we adopted for complete remission (pain score of ≤2, no further medication prescribed) is possibly another reason for lower remission rates, since other studies have defined a pain-free state with an NRS score of less than 3, or without discussion of medication withdrawal [8, 25].

Epidural hematoma, infection, and abscess are the complications that make continuous epidural catheterization difficult, but no infections were reported after continuous epidural infusion in this study. This is likely due to the involvement of well-trained physicians who changed dressings daily and well-educated patients and caregivers. The incidence of epidural hematoma is low and was not observed in the present study. However, one patient experienced severe urinary retention after the procedure, which was resolved after the epidural catheter was removed [1].

### Limitations

First, this was a retrospective study, and there may be an influence of unmeasured confounding variables. Thus, we conducted a covariance analysis with the baseline demographics and underlying patient disease as covariates to control for potential disturbance factors. Additionally, only the patients who took both anticonvulsants and analgesics along with continuous epidural infusion were included in the study to ensure consistent drug use across the sample set.

Second, our research data were derived from electronic medical records, which may have led to an underestimation of the actual incidence of side effects. In the present study, continuous epidural infusion was discontinued in only one patient because of adverse effects, but side effects such as dysuria and motor weakness may not have been added to the medical record when the epidural block was maintained because of low symptom severity.

Third, we excluded patients who were treated with other interventional procedures within the 6-month period, and this could have caused a selection bias in the study. Nevertheless, if we had included patients who experienced other interventions in the analysis, there would have been uncertainty regarding whether the patient symptoms improved due to receiving a continuous epidural infusion for the first time, or because they had other interventions. Therefore, we excluded patients with other interventions when calculating complete remission and 6-month pain scores and analyzed the ratios separately.

Fourth, we investigated whether oral medications were administered simultaneously with continuous epidural infusion for herpes zoster, but the exact doses were not measured. The correct dose of oral medication may affect the incidence of PHN and pain in herpes zoster, which may limit the results of this study.

## Conclusion

Continuous epidural catheterization combined with standard drug therapy in patients with acute herpes zoster may be effective in preventing the associated pain and development of PHN. Furthermore, using electric stimulation to identify the specific epidural location affected by the herpes zoster infection and administering the drug via an epidural catheter enables continuous drug administration to the exact site of neurological damage. A well-planned, prospective study comparing the methods for preventing herpes zoster-related pain and PHN is required to validate the results of the present study.

## Data Availability

The data analyzed during the current study are available from the corresponding author upon reasonable request.

## Abbreviations

NRS: Numeric rating scale
PHN: Postherpetic neuralgia
VZV: Varicella zoster virus
